# Allicin ameliorates sepsis-induced acute kidney injury through Nrf2/HO-1 signaling pathway

**DOI:** 10.1007/s11418-023-01745-3

**Published:** 2023-09-05

**Authors:** Xiao-Jun Li, Ting Liu, Yuan Wang

**Affiliations:** 1 Department of Nephrology, The Second Hospital of Dalian Medical University, 467 Zhongshan Road, Dalian, 116027 Liaoning China; 2 Department of General Practice, The Second Hospital of Dalian Medical University, 467 Zhongshan Road, Dalian, 116027 Liaoning China

**Keywords:** Sepsis, Acute kidney injury, Allicin, Nrf2, HO-1

## Abstract

Acute kidney injury (AKI) is a complication that can be induced by different factors. Allicin is a class of organic sulfur compounds with anticancer and antibacterial effects, and has not been reported in sepsis-induced AKI (S-AKI). S-AKI was induced in c57BL/6 mice by cecal ligation puncture. In response to the treatment of allicin, the survival rate of mice with S-AKI was increased. Reduced levels of serum creatinine, blood urea nitrogen, UALB, KIM-1 and NGAL indicated an improvement in renal function of S-AKI mice. Allicin inhibited the inflammation and cell apoptosis, which evidenced by decreased levels of inflammatory cytokines and apoptosis-related proteins. Oxidative stress was evaluated by the levels of oxidative stress biomarkers, and suppressed by allicin. In addition, allicin-alleviated mitochondrial dysfunction was characterized by decreased JC-1 green monomer. These effects of allicin were also evidenced in HK2 cells primed with lipopolysaccharide (LPS). Both in vivo and in vitro experiments showed that the nuclear translocation of Nrf2 and the expression of HO-1 increased after allicin treatment, which was confirmed by ML385 and CDDO-Me. In summary, this study revealed the alleviating effect of allicin on S-AKI and demonstrated the promotive effect of allicin on nuclear translocation of Nrf2 for the first time. It was inferred that allicin inhibited the progression of S-AKI through Nrf2/HO-1 signaling pathway. This study makes contributions to the understanding of the roles of allicin in S-AKI.

**Supplementary Information:**

The online version contains supplementary material available at 10.1007/s11418-023-01745-3.

## Introduction

Sepsis is a systemic inflammatory response syndrome caused by dysregulated host response to infection. It is a major cause of multiple organ failure, septic shock and death [1]. The in-hospital mortality rate of sepsis is 20–30% [2, 3]. Acute kidney injury (AKI) is one of the most common organ failure symptoms caused by sepsis. More than three-fifths of patients with sepsis developed AKI [4], and the mortality of patients with sepsis-induced AKI (S-AKI) is very high [5]. Increased serum creatinine (Scr) levels and/or decreased urine production are clinical manifestations of AKI [6]. Researches on S-AKI are extensive, but the pathogenic mechanism of S-AKI is still not completely clear. Based on the current research background, the pathological mechanism of S-AKI may be related to microcirculation dysfunction, inflammation and adaptive response of renal tubular epithelial cells to harmful signals [7]. Current treatments of S-AKI are mainly the use of antibiotics and drainage for source control and organ support [8]. Therefore, the development and innovation of therapeutic means and drugs to effectively alleviate S-AKI still need more research support.

Allicin (*Diallyl thiosulfinate*) is an organic sulfur compound extracted from garlic (*Allium sativum*), which is one of the most important economic plants and ranks second with onions among the most widely used *Allium* species in the world [9]. Allicin was first extracted by Cavallito and Bailey in 1944 and has been extensively studied since then [10]. It possesses antibacterial, anticancer and anti-inflammatory activities [11]. The antimicrobial effects of allicin against *Helicobacter pylori, Bacillus aureus, Staphylococcus aureus* and *Candida albicans* have been reported in several studies [12]. In terms of anticancer, allicin has important implications for digestive system cancers, including gastric, colorectal, liver, bile duct, and pancreatic cancers [13]. Allicin has been found to play a positive role in numerous inflammatory diseases, such as ankylosing spondylitis [14], osteoarthritis [15] and ulcerative colitis [16]. In addition, allicin attenuates acute lung injury caused by sepsis [17, 18] and acute hepatitis [19]. However, the role of allicin in S-AKI remains unclear. Studies found that allicin delayed the progression of diabetic nephropathy by inhibiting oxidative stress and inflammation [20–22]. Allicin exerted nephroprotective effects by inhibiting apoptosis in trauma/hemorrhagic shock [23]. We speculate that allicin may also have renoprotective effects on S-AKI.

Nuclear factor erythroid 2-related factor 2 (Nrf2), an important transcription factor regulating heme oxygenase-1 (HO-1), is degraded by Kelch-like ECH-associated protein (Keap1) under normal circumstances. In the event of injury, Nrf2 is released from Nrf2-Keap1 complex, then translocated to the nucleus [24]. Activation of the Nrf2/HO-1 pathway is known to reduce tissue inflammation, oxidative stress, and apoptosis, thereby alleviating sepsis-induced failure of multiple organs, including lung, kidney, and liver [25, 26]. Therefore, we speculate that allicin may exert anti-inflammatory, anti-oxidative stress and anti-apoptotic effects in S-AKI by regulating the Nrf2/HO-1 pathway. This paper intends to carry out animal and cell experiments to explore the role and mechanism of allicin in S-AKI.

## Materials and methods

### Animal and treatment

Cecal ligation puncture (CLP) was used to induce sepsis in mice [17, 27]. C57BL/6 male mice aged 8 weeks were randomly divided into 5 groups: Sham, CLP, CLP + LA, CLP + MA and CLP + HA. Considering the 3R principle of animal research, and the lack of suitable positive control drugs of the disease, we gave up the more perfect experimental scheme of setting a positive control group and a normal group. Following anesthesia, the abdomen of the mice was shaved and sterilized. A scalpel was used to make a 1.5–2 cm longitudinal incision along the midline of the skin. The cecum was isolated and ligated at half the distance between distal pole and the base of cecum without disrupting or damaging the mesenteric vessels. The cecum was punctured with needle and then expressed a small amount of fecal material with a gentle squeeze. After the cecum was returned to the abdominal cavity, the peritoneum and muscle were closed in separate layers, and the skin was sutured at last. In Sham group, cecal ligation and perforation were not performed, but other operations were consistent with CLP group. Mice in the treatment group were intraperitoneally injected with different doses of allicin (Sigma, MO, USA) at 1 h after operation. CLP + LA group: 5 mg/kg allicin (Yuanye, Shanghai, China) every 12 h, CLP + MA group: 10 mg/kg allicin every 12 h, CLP + HA group: 20 mg/kg allicin every 12 h. The Sham group and CLP group were given the same volume of normal saline in the same way. After 24 h, some mice were killed, and renal cortex, urine and serum samples were collected for subsequent experiments. The remaining mice were monitored for survival analysis.

### Cell culture

HK2 cell line was purchased from Procell Life Science & Technology (Wuhan, China), and it was cultured in MEM (Solarbio, Beijing, China) supplemented with 10% fetal bovine serum (Tianhang, Zhejiang, China) in an incubator at 37 °C with 5% CO_2_. To explore the effect of allicin on LPS-induced injury, cells were treated with 20 μg/mL allicin and 1 μg/mL LPS (Solarbio) for 24 h. To verify the effect of allicin on Nrf2/HO-1 signal pathway, some cells were treated with allicin (20 μg/mL) and LPS (1 μg/mL) and ML385 (Yuanye) (10 μm) for 24 h, and others were treated with allicin (20 μg/mL) and LPS (1 μg/mL) and CDDO-Me (4 µmol/L) for 24 h.

### Assessment of kidney function and analysis of oxidative stress markers

Scr and blood urea nitrogen (BUN) levels were measured to assess kidney function. Creatinine assay kit uses enzymatic assay to detect Scr. Urea assay kit was used to determine BUN. Detection of oxidative stress markers in renal cortex and HK2 cells: Reactive oxygen species (ROS) level was measured by chemifluorescence using a ROS assay kit. Glutathione peroxidase (GSH-Px) was detected by GSH-Px assay kit according to the manufacturer’s instructions. Malondialdehyde (MDA) and superoxide dismutase (SOD) were examined by MDA assay kit and SOD assay kit, respectively. All kits were purchased from Nanjing Jiancheng Bioengineering Institute (Nanjing, China).

### Enzyme-linked immunosorbent assay (ELISA)

Following centrifugation of urine for 20 min, the supernatant was collected for ELISA assay. Mouse Urinary Albumin (UALB) ELISA Kit was used to measure the UALB. Urine neutrophil gelatinase-associated lipocalin (NGAL) was detected by Mouse NGAL ELISA Kit. We performed the detection of urinary kidney injury molecule 1 (KIM-1) using mouse KIM-1 ELISA Kit. The three ELISA kits were purchased from Wuhan Fine Biotech (Wuhan, China).

The levels of interleukin-6 (IL-6), IL-1β, tumor necrosis factor alpha (TNF-α) and monocyte chemoattractant protein-1 (MCP-1) in renal cortex and HK2 cells were detected according to ELISA kit instructions. The kits used in this part were purchased from MULTI SCIENCES (Zhejiang, China) and they included Mouse IL-6 ELISA kit, Human IL-6 ELISA kit, mouse IL-1β ELISA kit, human IL-1β ELISA kit, mouse TNF-α ELISA kit, human TNF-α ELISA kit, mouse MCP-1 ELISA kit and Human MCP-1 ELISA Kit.

### CCK-8

HK2 cells were seeded in a 96-well culture plate with 4 × 10^3^ cells per well for 12 h culture. Cells were treated with different doses of allicin (0, 5, 10, 20, 40 and 80 μg/mL) for 24 h and evaluated using the CCK-8 (Beyotime, Shanghai, China). Cells treated with allicin at various concentrations (0, 5, 10, 20, 40 and 80 μg/mL) and 1 μg/mL LPS for 24 h were measured by CCK-8 assay. Cells in each well were treated with 10 μl CCK-8 and then cultured in 5% CO_2_ at 37 °C for 2 h, followed by detection of the absorbance value (OD) at 450 nm.

### TUNEL staining

In Situ Cell Death Detection Kit, TMP red (Roche, Switzerland) was used for TUNEL staining to evaluate the renal cortical cell apoptosis. DAPI staining was performed to distinguish the non-apoptotic cells. Stained sections were observed under a BX53 microscope (Olympus, Tokyo, Japan) and photographed using DP73 camera (Olympus).

### Hematoxylin–eosin (HE) staining

HE staining was performed to observe the histopathological changes in kidney tissues. The dehydrated renal cortical tissue was embedded in paraffin and cut into 5 μm sections, which were stained with hematoxylin and eosin. The staining was observed under a BX53 microscope.

### Immunohistochemistry (IHC)

IHC staining for cleaved caspase-3 was used to evaluate apoptosis of renal cortical cells. The kidney sections of mice were deparaffinized and rehydrated, followed by an antigen retrieval procedure. Slides were incubated with 3% hydrogen peroxide for 15 min to eliminate endogenous peroxidase activity and then blocked with 1% BSA (Sangon, Shanghai, China). Primary antibody against to cleaved caspase-3 (AF7022, Affinity, Jiangsu, China) was diluted in PBS at 1:100. Secondary antibody HRP-labeled goat anti-rabbit IgG (31460, Thermo Fisher, PA, USA) was diluted in PBS at 1:500. The sections were incubated with primary antibody overnight at 4 °C and secondary antibody for 1 h at 37 °C. After staining with DAB and hematoxylin, the sections were observed under a microscope and representative pictures were selected.

### Immunofluorescence (IF)

Cells were fixed with 4% paraformaldehyde for 15 min. Next, Triton X-100 (0.1%) was used to incubate cells for 30 min. Cell block was conducted with 1% BSA for 15 min. All these operations were performed at room temperature. Primary antibody incubation was performed with Nrf2 antibody (1:100 diluted in PBS) (AF0639, Affinity) overnight at 4 °C. After washing three times with PBS, cells were incubated with Cy3-conjugated goat anti-rabbit IgG (1:200 diluted in PBS) (ab6939, Abcam, UK) for 1 h at room temperature in a dark environment. Finally, nuclei were stained with DAPI. Staining was observed under a BX53 microscope (Olympus).

### Western blot (WB)

RIPA lysate buffer (Solarbio) was used to extract total protein from kidney tissue and HK2 cells. Mitochondrial protein was extracted using mitochondrial protein extraction kit (Nanjing Jiancheng). Nuclear protein extraction kit (Solarbio) was used to separate nuclear and plasma proteins. The protein concentration was determined using the BCA protein concentration assay kit (Solarbio). Samples containing equal amounts of proteins were separated by SDS-PAGE and the separated proteins were transferred to PVDF membranes which were re-wetted with 100% methanol. Subsequently, the membranes were blocked with 5% skim milk and incubated with the corresponding antibodies overnight at 4 °C. The primary antibodies were as follows: HO-1 antibody (1:1000 dilution) (AF5393, Affinity), Nrf2 antibody (1:1000 dilution) (AF0639, Affinity), cytochrome c antibody (1:1000 dilution) (A0225, ABclonal, Shanghai, China), Bcl-2 antibody (1:500 dilution) (A0208, ABclonal), Bax antibody (1:500 dilution) (A19684, ABclonal), cleaved caspase-9 antibody (1:1000 dilution) (AF5240, Affinity), cleaved caspase-3 antibody (1:1000 dilution) (AF7022, Affinity), Histone H3 antibody (1:5000 dilution) (GTX122148, Gene Tex, TX, USA), COX IV antibody (1:1000 dilution) (GTX49132, Gene Tex), β-actin antibody (1:10000 dilution) (66009–1-Ig, Proteintech, IL, USA). The samples incubated with COX IV and β-actin antibody were incubated with goat anti-mouse IgG-HRP (SE131, Solarbio). Samples incubated with other antibodies were incubated with goat anti-rabbit IgG-HRP (SE134, Solarbio) at 37 °C for 1 h. The secondary antibodies were diluted at 1:3000. Finally, the target protein was detected by enhanced chemiluminescent detective system.

### Flow cytometry

HK2 cells (5 × 10^5^) were seeded in 6-well plate and treated with reagents (see section: Cell culture). Centrifugation at 150 g for 5 min was conducted to collect the cells of each group. After washing twice with PBS, cells were re-suspended with 500 μl binding buffer and stained with 5 μl AnnexinV-FITC and 5 μl PropidiumIodide (KeyGen, Nanjing, China) for 10 min at room temperature in the dark, followed by detection using a flow cytometer (NovoCyte, ACEA, CA, USA). To detect mitochondrial membrane potential, cells were stained with JC-1 (KeyGen) for 20 min in an incubator at 37 °C with 5% CO_2_, then washed twice with incubation buffer (1 ×). The cell pellets were re-suspended with 500 μl incubation buffer (1 ×). The mitochondrial membrane potential of the cells was detected by flow cytometry.

### Statistical analysis

Statistical analysis was carried out using GraphPad Prism 8.0.2 software (USA). TUNEL and IHC staining signals were quantified by Image J software. Survival curves of mice were generated by Kaplan–Meier method and survival difference was determined by log-rank (Mantel-Cox) test. Ordinary one-way ANOVA and unpaired t test were used to compare the means in different groups. Differences were considered significant at *p* < 0.05.

## Results

### Allicin relieved S-AKI induced by CLP

**Fig. 1:**
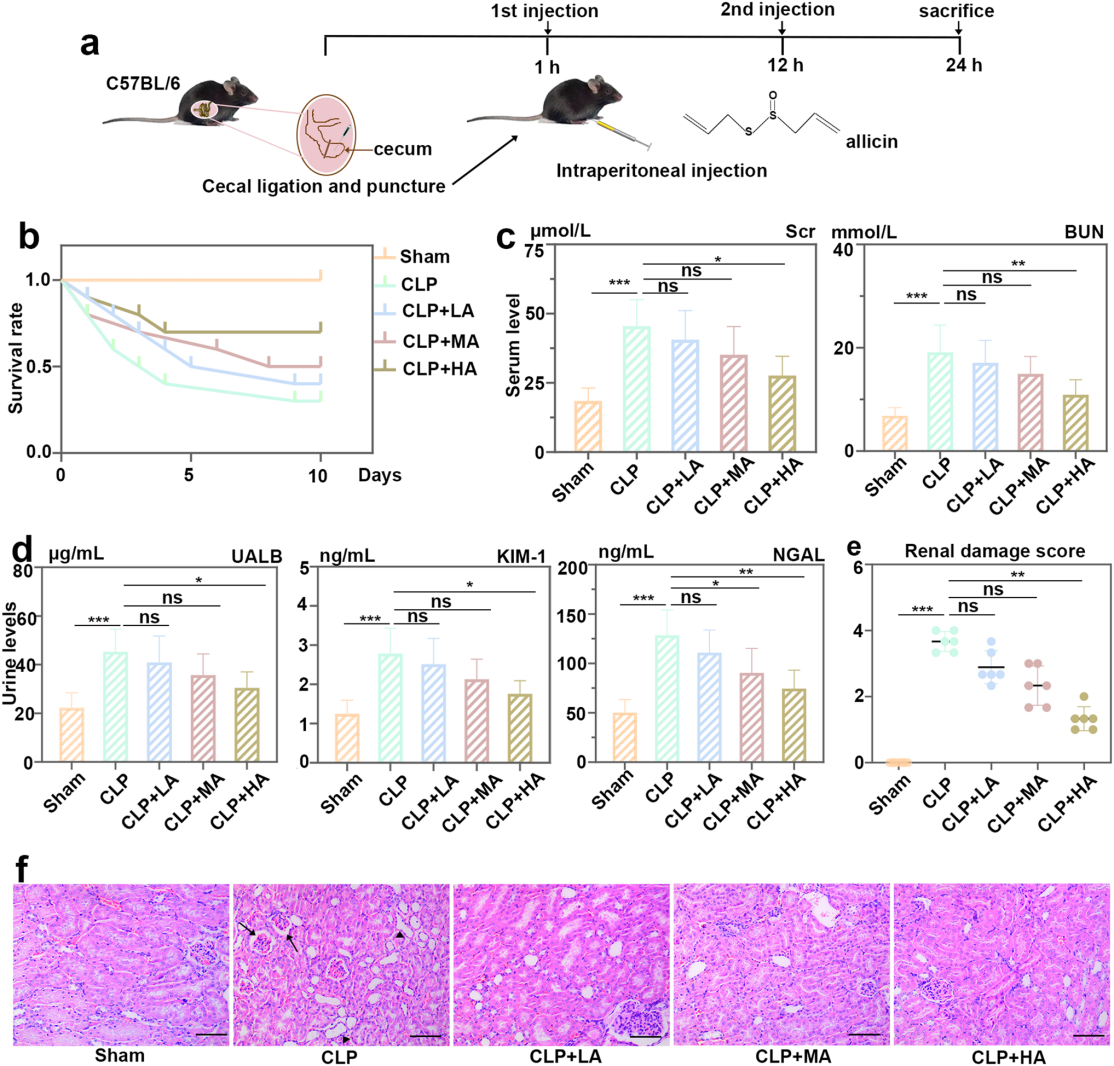
Allicin relieved acute kidney injury (AKI) induced by cecal ligation puncture (CLP). **a** Schematic diagram of the experimental protocol for the CLP-induced mouse model of sepsis. **b** Survival of mice treated with allicin after CLP, n = 10. **c** Levels of serum creatinine (Scr) and blood urea nitrogen (BUN) in mice with treatments of CLP and allicin. **d** Levels of urine albumin (UALB), kidney injury molecule 1 (Kim-1) and neutrophil gelatinase-associated lipocalin (NGAL) in allicin-treated mice of CLP-induced sepsis. **e** Renal damage score based on HE staining. **f** HE staining of kidney cortex tissue in experimental mice, black arrows represent glomerular atrophy, and triangles represent diffuse dilation of renal tubules, bar = 100 μm. All data were expressed as mean ± SD. “ns”: no significance, * *p* < 0.05, ** *p* < 0.01, and *** *p* < 0.001, *n* = 6 in each group, and the number of technical replicates is 3

The mice were induced with sepsis by CLP. Kaplan–Meier survival analysis was conducted to evaluate the 10-day survival rates of mice in the five groups (Fig. 1a). Allicin-treated mice had an increased survival rate compared with mice treated with CLP. Mice treated with 20 mg/kg allicin showed the highest survival rate (Fig. 1b). Serum Scr and BUN levels in mice were increased after CLP, but significantly decreased after allicin (20 mg/kg) injection (Fig. 1c). To further evaluate renal injury, the protein levels of albumin, KIM-1 and NGAL in the urine were detected by ELISA, it showed that allicin at a dose of 20 mg/kg inhibited the expression of albumin, KIM-1 and allicin at a dose of 10 mg/kg decreased NGAL level (Fig. 1d). The renal injury was observed by HE staining. According to the scoring criteria of kidney injury reported by previous literatures [28], we assessed kidney injury and presented the results in Fig. 1e. Histological scoring was done by two trained investigators in a blind fashion. It was found that the kidney tissue in the operation group had obvious glomerular atrophy and diffuse expansion of renal tubules. Symptoms of kidney tissue were alleviated after allicin treatment (Fig. 1f). These results proved the successful establishment of the septic mouse model. In conclusion, allicin at a dose of 20 mg/kg effectively relieved CLP-induced S-AKI.

### Allicin suppressed inflammatory responses and oxidative stress, and ameliorated mitochondrial dysfunction in AKI

**Fig. 2:**
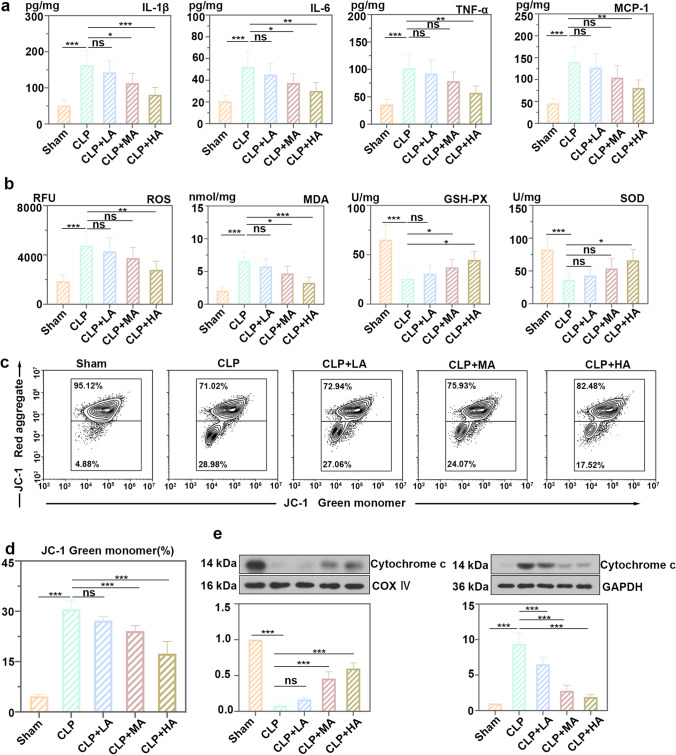
Allicin suppressed inflammatory responses and oxidative stress, and ameliorated mitochondrial dysfunction in AKI. **a** Levels of cytokines interleukin-6 (IL-6), IL-1β, tumor necrosis factor alpha (TNF-α) and monocyte chemoattractant protein-1 (MCP-1) in the kidneys from experimental mice. **b** Levels of reactive oxygen species (ROS) and malondialdehyde (MDA), as well as activities of glutathione peroxidase (GSH-Px) and superoxide dismutase (SOD) in kidney of mice treated with allicin after CLP. **c** Analysis of JC-1-labeled mitochondrial membrane potential changes by flow cytometry in kidney cortex. **d** Analysis of JC-1 green monomer of Fig. 2c. **e** Protein levels of cytochrome c in mitochondria and cytoplasm of renal cortical cells. All data were expressed as mean ± SD. “ns”: no significance, * *p* < 0.05, ** *p* < 0.01, and *** *p* < 0.001, *n* = 6 in each group, and the number of technical replicates is 3

The protein levels of IL-6, IL-1β, TNF-α, and MCP-1 in the renal cortex of allicin-treated mice were decreased compared with those in the CLP group (Fig. 2a), which demonstrated that allicin suppressed inflammation. Oxidative stress markers detected in this study include ROS, SOD, GSH-Px, and MDA. Among them, the levels of ROS and MDA increased in the CLP group and decreased after allicin treatment. The activities of SOD and GSH-Px were enhanced in the group with allicin treatment (Fig. 2b). Cells from kidney cortex were dyed with JC-1 to determine the mitochondrial membrane potential. Mitochondria with high membrane potential accumulates JC-1, which emits red fluorescence. As the allicin concentration increased, the numbers of cells with red fluorescence increased (Fig. 2c and d). The release of cytochrome C from mitochondria to the cytoplasm reduced by allicin (Fig. 2e), it turned out that allicin increased the mitochondrial membrane potential and ameliorated mitochondrial dysfunction.

### Allicin ameliorated renal injury through Nrf2/HO-1 signaling pathway

**Fig. 3:**
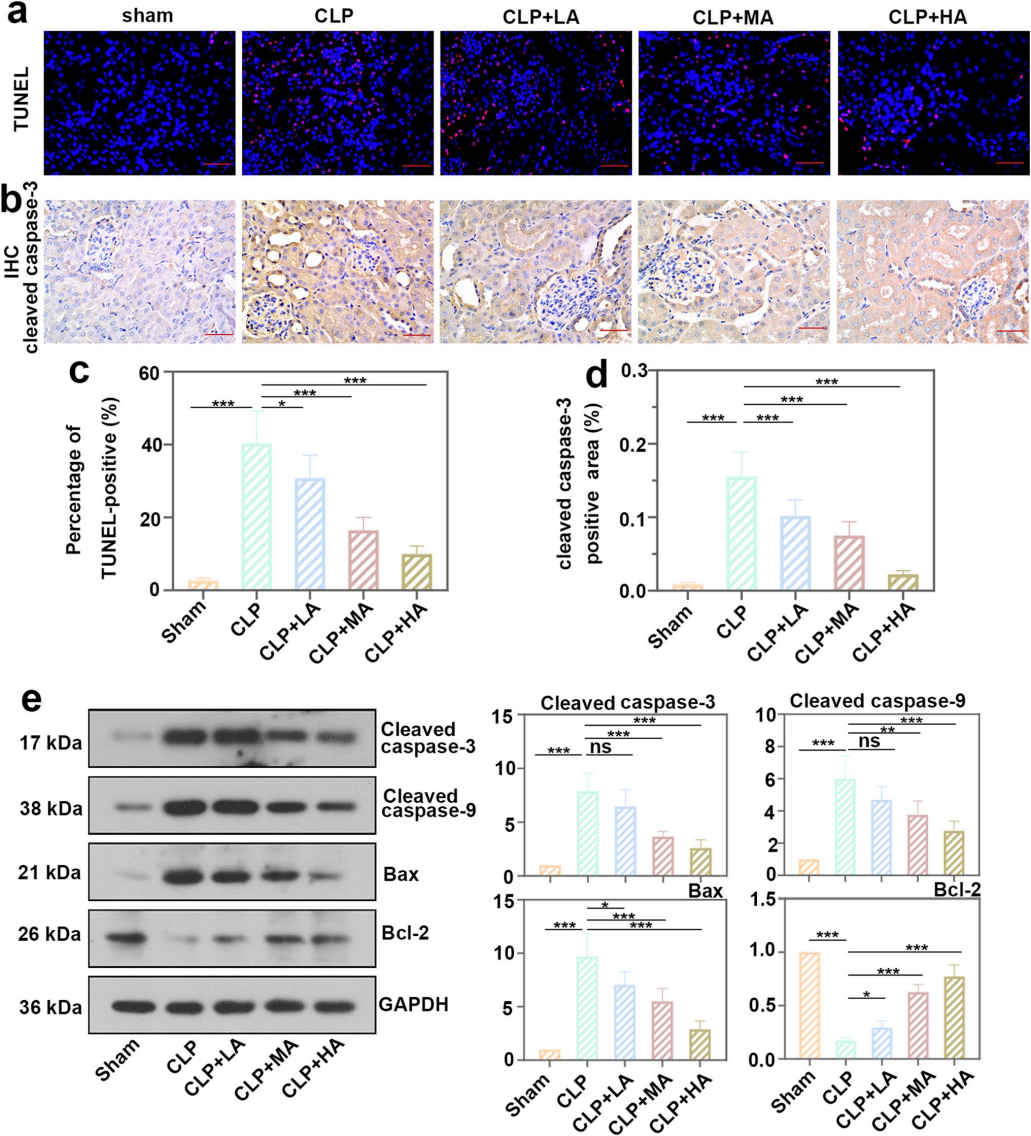
Allicin ameliorated renal injury through Nrf2/HO-1 signaling pathway. **a** TUNEL staining of kidney cortex tissue, bar = 50 μm. **b** IHC staining of cleaved caspase-3 in kidney tissue of mice with allicin and CLP treatment, bar = 50 μm. **c** The percentage of TUNEL-positive (dead) cells. **d** Cleaved caspase-3 positive area (%) in IHC staining. **e** Protein levels of cleaved caspase-3, cleaved caspase-9, Bax, and Bcl-2 in kidney cortex tissue of experimental mice. All data were expressed as mean ± SD. “ns”: no significance, * *p* < 0.05, ** *p* < 0.01, and *** *p* < 0.001, *n* = 6 in each group, and the number of technical replicates is 3

**Fig. 4:**
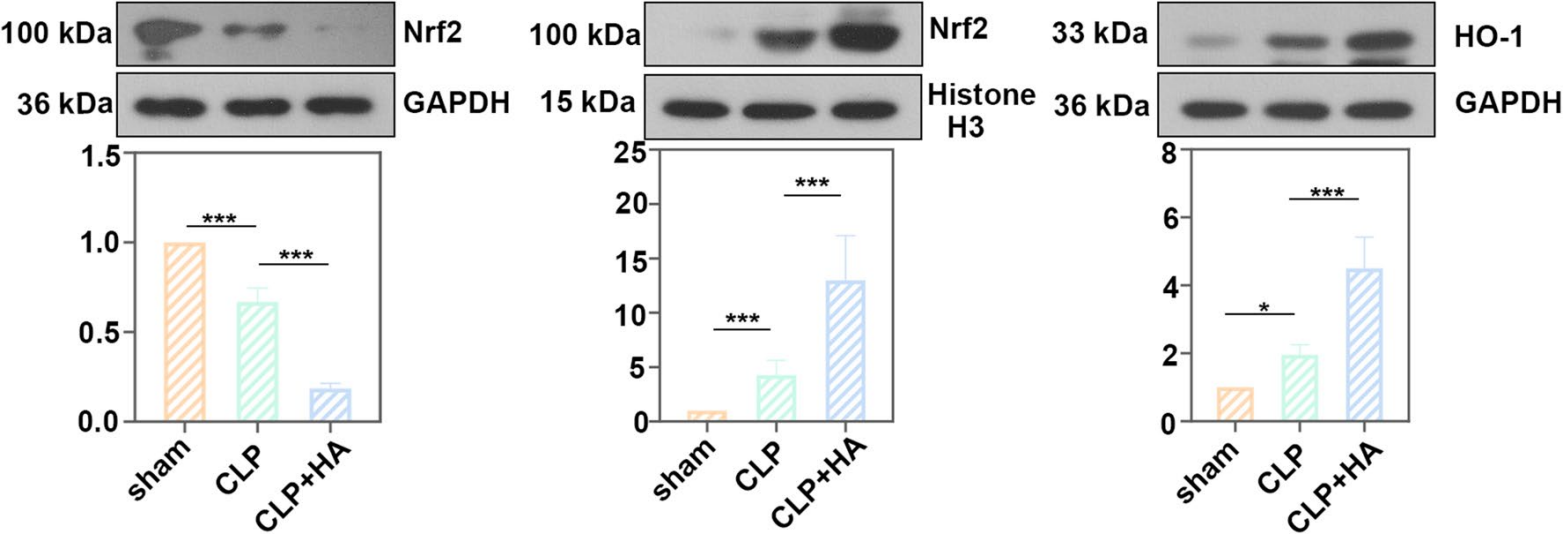
Protein levels of Nrf2 and HO-1. Protein levels of nuclear factor erythroid 2-related factor 2 (Nrf2) and heme oxygenase-1 (HO-1) in renal cortical cells of the mice. All data were expressed as mean ± SD. “ns”: no significance, * *p* < 0.05 and *** *p* < 0.001, *n* = 6 in each group, and the number of technical replicates is 3

TUNEL staining showed that allicin reduced the number of apoptotic cells in renal cortex (Fig. 3a and c). Representative images of IHC staining for cleaved caspase-3 in renal cortex showed that positive staining was reduced in the administration group compared with the operation group (Fig. 3b and d). The protein levels of cleaved caspase-3, cleaved caspase-9, and Bax were decreased in the renal cortex after allicin treatment. The level of anti-apoptotic protein Bcl-2 was increased compared with the operation group (Fig. 3e). It proved that allicin inhibited apoptosis in AKI. WB found that the protein level of Nrf2 was decreased in the cytoplasm and increased in the nucleus in the allicin-treated group (Fig. 4). It demonstrated that allicin promoted the nuclear translocation of Nrf2. There was an elevation of HO-1 level of in cells treated with allicin (Fig. 4). Nrf2/HO-1 pathway plays important roles in anti-oxidation and anti-inflammation. Combined with the results of the first two parts, it further showed that allicin is likely to promote the Nrf2/HO-1 signaling pathway to suppress AKI.

### Allicin inhibited the inflammatory response and oxidative stress, and restored mitochondrial function in HK2 cells primed with LPS

**Fig. 5:**
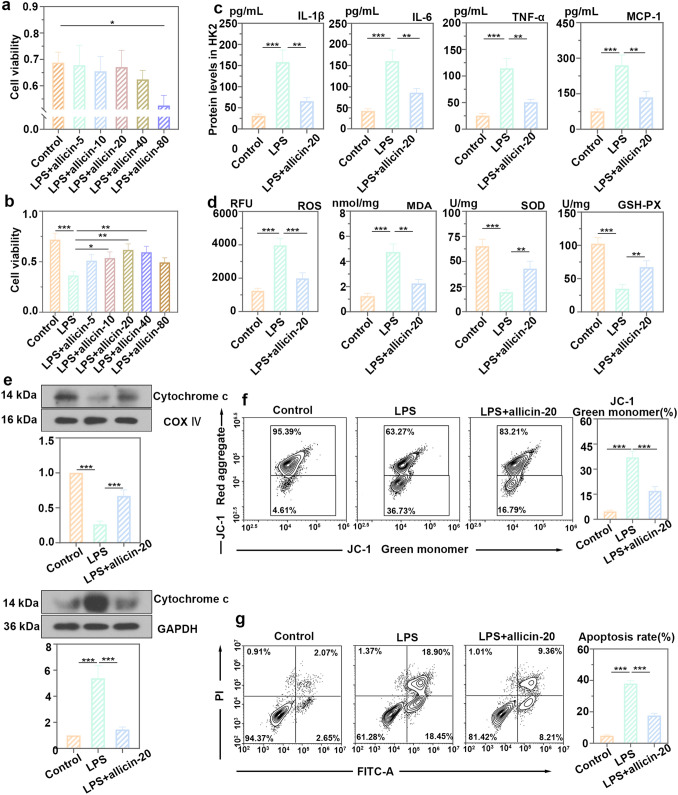
Allicin inhibited the inflammatory response and oxidative stress, and restored mitochondrial function in HK2 cells primed with lipopolysaccharide (LPS). **a** CCK-8 analysis of the viability of HK2 cells treated with allicin. **b** CCK-8 analysis of the viability of HK2 cells with LPS and allicin treatments. **c** Protein levels of IL-6, IL-1β, TNF-α and MCP-1 in HK2 cells with LPS and allicin treatments. **d** Levels of ROS and MDA, as well as activities of SOD and GSH-Px in LPS-primed HK2 cells after allicin treatment. **e** Levels of cytochrome c in the mitochondria and cytoplasm of HK2 cells treated with LPS and allicin. **f** Analysis of JC-1-labeled mitochondrial membrane potential changes by flow cytometry in HK2 cells, and statistical analysis of JC-1 green monomer. **g** Detection of cell apoptosis by flow cytometry in HK2 cells with LPS and allicin treatment. All data were expressed as mean ± SD. “ns”: no significance, ** *p* < 0.01, and *** *p* < 0.001, *n* = 3 in each group, and the number of technical replicates is 3

To determine the effect of allicin on the viability of HK2 cells, HK2 cells were treated with gradient concentrations of allicin. It was found that the viability of HK2 cells with the treatment of allicin at a dose between 5 mg/kg and 40 mg/kg had no significant difference compared with the control group (Fig. 5a). HK2 cells primed with LPS were treated with a gradient concentration of allicin. The results showed that 10, 20 and 40 mg/kg allicin increased the viability of cells and the cell viability was strongest at a dose of 20 mg/kg allicin (Fig. 5b). Therefore, allicin at a concentration of 20 mg/kg was used to evaluate its effect on HK2 cells. ELISA showed that the protein levels of IL-6, IL-1β, TNF-α and MCP-1 were decreased in allicin group compared with LPS group (Fig. 5c). The levels of ROS and MDA were decreased in cells with allicin treatment, while the activities of GSH-Px and SOD were enhanced (Fig. 5d). It indicated that allicin at 20 mg/kg inhibited LPS-induced inflammatory response and oxidative stress. After allicin treatment, the release of mitochondrial cytochrome c into the cytoplasm was reduced (Fig. 5e). Analysis of JC-1-labeled mitochondrial membrane potential found that allicin-alleviated LPS-induced mitochondrial depolarization (Fig. 5f). In addition, LPS-induced cell apoptosis was inhibited by allicin (Fig. 5g).

### Allicin inhibited the cytotoxicity of LPS in HK2 cells by promoting the Nrf2/HO-1 signaling pathway

**Fig. 6:**
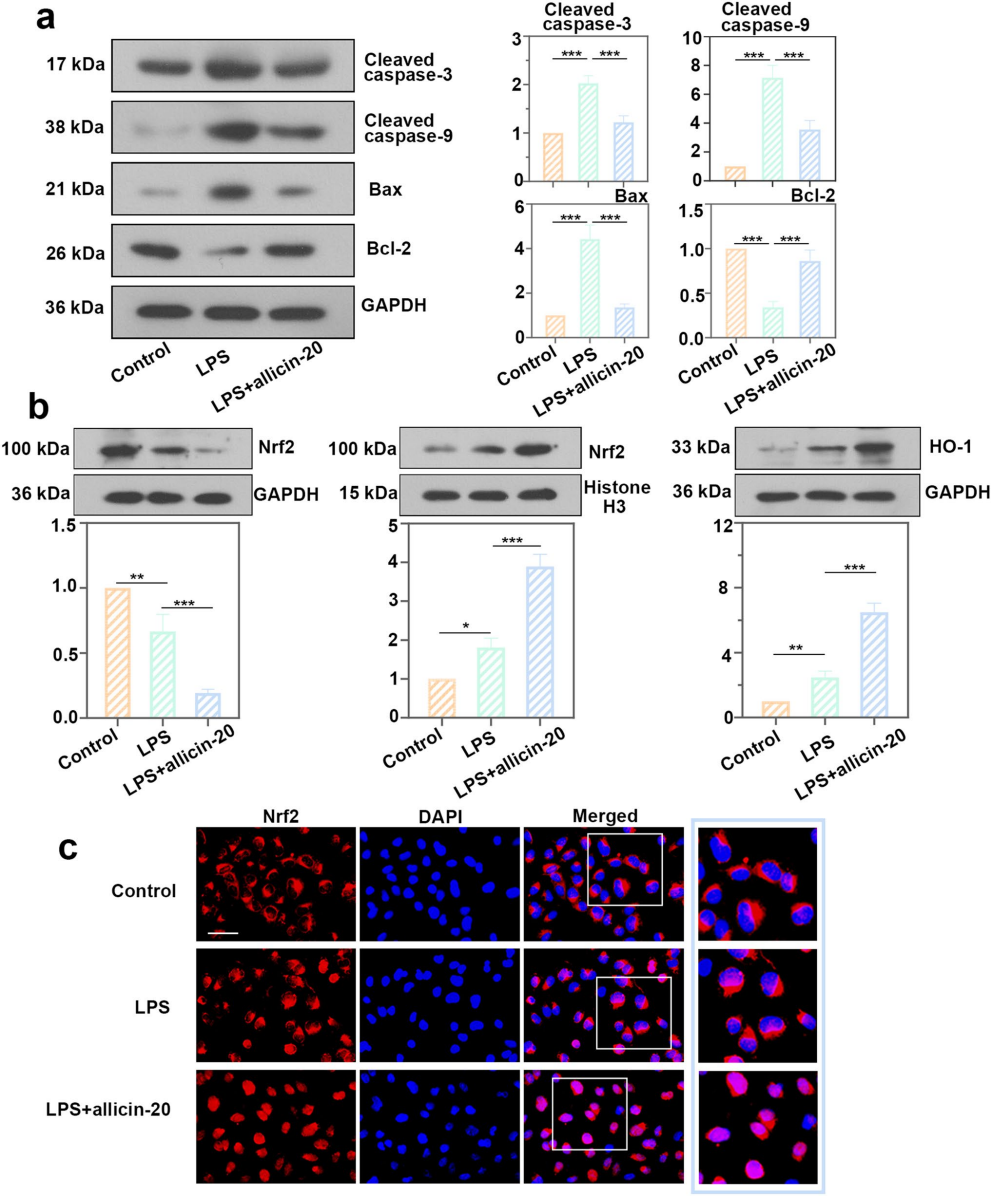
Allicin inhibited the cytotoxicity of LPS in HK2 cells by promoting the Nrf2/HO-1 signaling pathway. **a** Protein levels of cleaved caspase-3, cleaved caspase-9, Bax, and Bcl-2 in HK2 cells after allicin and LPS treatment. **b** Protein levels of Nrf2 and HO-1 in HK2 cells treated with allicin and LPS. All data were expressed as mean ± SD. **c** The accumulation of NRF2 in the nucleus was detected by immunofluorescence, bar = 50 μm. * p < 0.05, ** *p* < 0.01, and *** *p* < 0.001, *n* = 3 in each group, and the number of technical replicates is 3

**Fig. 7:**
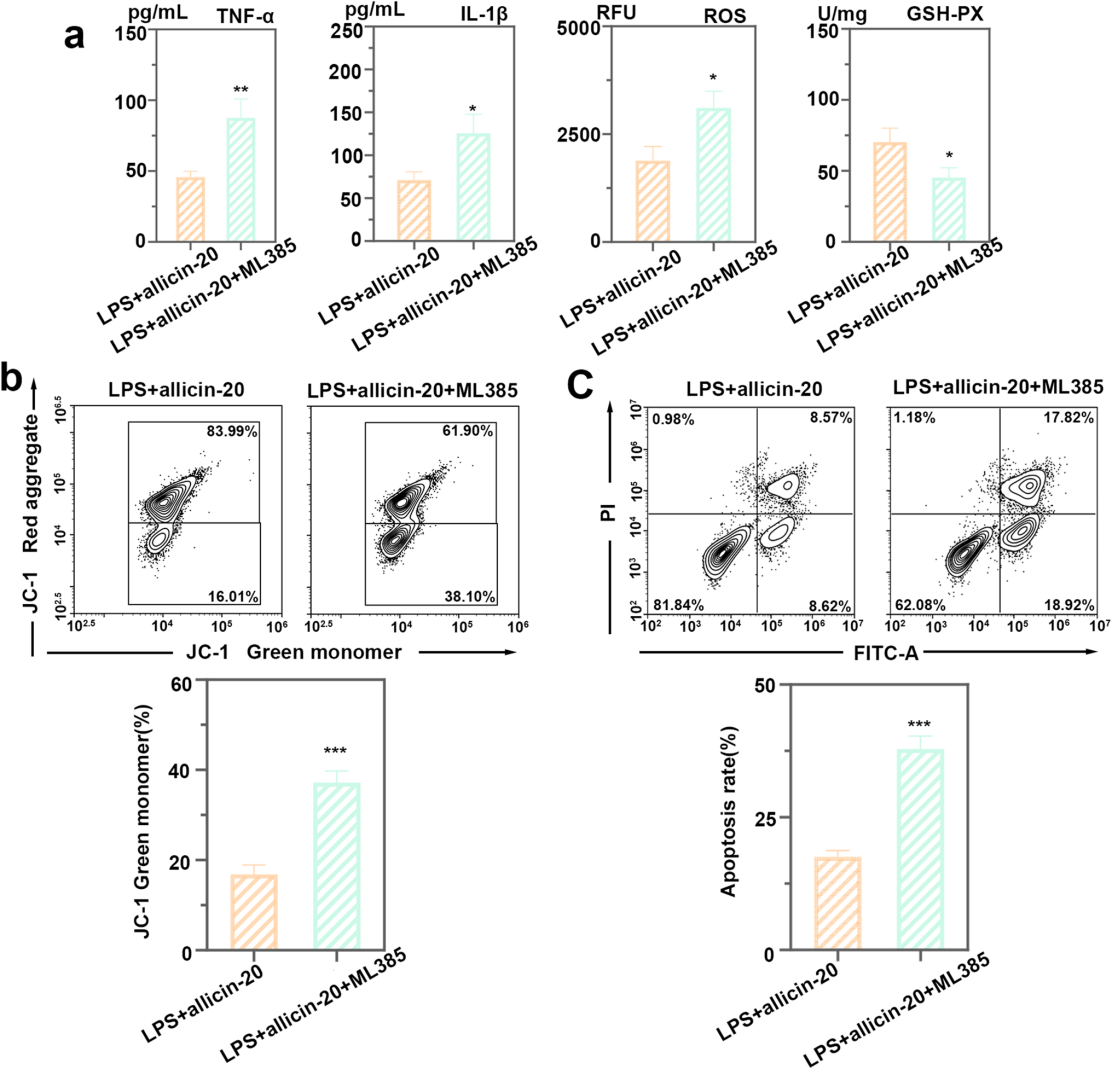
Validation of the promotive effect of allicin on Nrf2/HO-1 signaling pathway. **a** Levels of TNF-α, IL-1β and ROS as well as the activity of GSH-Px in HK2 cells with allicin and ML385 treatment. **b** Analysis of JC-1-labeled mitochondrial membrane potential by flow cytometry in LPS and allicin and ML385 treated HK2 cells. **c** Evaluation of cell apoptosis by flow cytometry in HK2 cells with LPS and allicin and ML385 treatments. All data were expressed as mean ± SD. * *p* < 0.05, ** *p* < 0.01, and *** *p* < 0.001, it was compared with LPS + allicin-20 group, *n* = 3 in each group, and the number of technical replicates is 3

Intracellular cleaved caspase-3, cleaved caspase-9, and Bax levels were inhibited by allicin, but Bcl-2 protein levels were increased (Fig. 6a). Allicin inhibited LPS-induced apoptosis. In addition, allicin treatment increased the nuclear translocation of Nrf2 and the expression of HO-1 in HK2 cells (Fig. 6b). We directly observed the accumulation of Nrf2 in the nucleus of the allicin group by immunofluorescence (Fig. 6c). ML385, an inhibitor of Nrf2, can inhibit the expression of Nrf2 downstream genes. To verify the promoting effect of allicin on Nrf2/HO-1 signaling pathway, HK2 cells were treated with ML385 and allicin. We found that the protein levels of inflammatory cytokines IL-6 and TNF-α and the level of ROS were increased after treatment with ML385, while the activity of GSH-Px was inhibited by ML385 (Fig. 7a). This demonstrated that the inhibitory effects of allicin on inflammation and oxidative stress were reversed by ML385. The treatment of ML385 promoted the mitochondrial depolarization (Fig. 7b). ML385 also promoted cell apoptosis of HK2 cells under the treatments of LPS and allicin (Fig. 7c). CDDO-Me, an agonist of Nrf2, was also used to verify the effect of allicin on Nrf2/HO-1 signal pathway (Supplementary figure). After treatment with 4 μmol/L CDDO-Me, the levels of TNF-α, IL-1β and ROS were decreased compared with LPS + allicin group (Supplementary figure a), in contrast, the level of GSH-PX was increased. Results of mitochondrial function analysis and cell apoptosis analysis were opposite to the results presented by ML385 treatment (Supplementary figure b and c). All above showed that CDDO-Me and allicin exhibited a synergistic effect on LPS-induced injury. Therefore, it was verified that allicin inhibited the cytotoxicity of LPS in HK2 cells by promoting the Nrf2/HO-1 signaling pathway.

## Discussion

AKI can be induced by a variety of factors. In addition to sepsis, others such as ischemia–reperfusion, rhabdomyolysis, SARS-CoV-2 (COVID19), food additives, serious scald injury, cancer therapy reagents and heavy metals can induce AKI [29–31]. The management of AKI is a major challenge. Plant extracts have been widely studied in the treatment of diseases because of their pharmacological activity and low side effects. In the research for therapeutic drugs for AKI, a variety of active molecules derived from plants have been reported, including ginkgetin aglycone [32], neferine [33], astragaloside IV [34], geniposide [35]. They have a significantly positive effect on reducing AKI. In this study, allicin from *Allium* plants was found to inhibit the progression of AKI. Although the above studies, including the present study, have validated the inhibitory activities of these plant extracts in AKI at the animal and cellular levels, there is a lack of clinical trials to further demonstrate their practical feasibility. This study only explored the inhibitory effect of allicin in S-AKI, whether it has a different effect in AKI induced by other incentives is still unknown, and it requires more research to clarify.

AKI is characterized by inflammation, increased oxidative stress, and mitochondrial dysfunction. In the search for potential drugs for AKI, the inhibitory effects on these responses need to be verified. Nrf2 is known to regulate genes related to antioxidant responses, inflammation and autophagy [36–38]. Related reports have confirmed that regulating Nrf2 and its downstream genes in the kidney reduced the release of inflammatory factors, inhibited oxidative stress and maintained cell viability [39]. HO-1 is a downstream target of Nrf2 with strong antioxidant, anti-inflammatory and anti-apoptotic properties [40, 41]. Many studies have used different models to verify that HO-1 mediated cytoprotection in AKI by regulating oxidative stress, inflammation and apoptosis [42]. Above studies demonstrate that Nrf2/HO-1 signaling pathway plays an inhibitory role in AKI. Macrophages are the main contributors of the inflammatory response of AKI and play an important role in the damage and repair process of AKI [43, 44]. Studies related to kidney diseases have shown that increased macrophage infiltration promoted kidney inflammation [45] and decreased macrophage polarization protected kidney function [46–48]. Interestingly, Nrf2/HO-1 signaling pathway has been shown to regulate the polarization and infiltration of macrophages [49]. These results again demonstrate the importance of Nrf2/HO-1 pathway in AKI.

Our study found that allicin could diminish the release of inflammatory factors, suppress oxidative stress and the release of cytochrome c from mitochondria to cytoplasm and apoptosis in renal injury induced by CLP and in HK2 cells treated with LPS. Furthermore, it found that allicin promoted the nuclear translocation of Nrf2, and the protein expression of downstream HO-1 was increased. Whether allicin targets Nrf2 and exerts the above-mentioned inhibitory effect by promoting Nrf2/HO-1 signaling pathway was confirmed by elimination of the inhibitory effect of allicin through ML385 and the synergistic effects of CDDO-Me and allicin. Therefore, we concluded that allicin relieved S-AKI through Nrf2/HO-1 signaling pathway. Allicin has been reported to activate nuclear translocation of Nrf2 in colon cancer studies [50], we found that allicin promoted the nuclear translocation of Nrf2 for the first time in S-AKI, however, the specific mechanism for regulation of Nrf2 by allicin has not been clearly reported.

Allicin has a variety of pharmacological activities as described in introduction, and several studies have reported its positive effects in anti-inflammatory, antiparasitic, immunomodulatory and renal protection [51]. The specific mechanism of allicin exerting these effects is closely related to its structure. Allicin is a kind of reactive sulfur species with oxidative activity [52]. Because of their hydrophobic properties, they easily cross cell membranes and subsequently oxidize mercaptans, such as cysteine residues in proteins and glutathione in cells, then change the protein structure, affect its function, and ultimately change the cell activity [11, 53]. Reversible oxidation and reduction of protein–mercaptan is central to the regulation of many cellular processes [54]. The nuclear translocation of Nrf2 involves the modification or oxidation of Keap1 to release Nrf2 from the complex. Keap1 contains a large number of cysteine residues in both humans and mice. At the same time, the changes of the three cysteine residues among them directly affect its conformation [55, 56]. Whether allicin oxidizes the cysteine residue of Keap1 has not been verified in this study. No studies have directly clarified whether allicin oxidizes Keap1. However, based on their structure and existing reports, allicin may interact with Keap1 and oxidize Keap1 to change its conformation, thus activating the nuclear translocation of Nrf2. This speculation is specifically raised here to encourage more researches on it.

**Fig.8:**
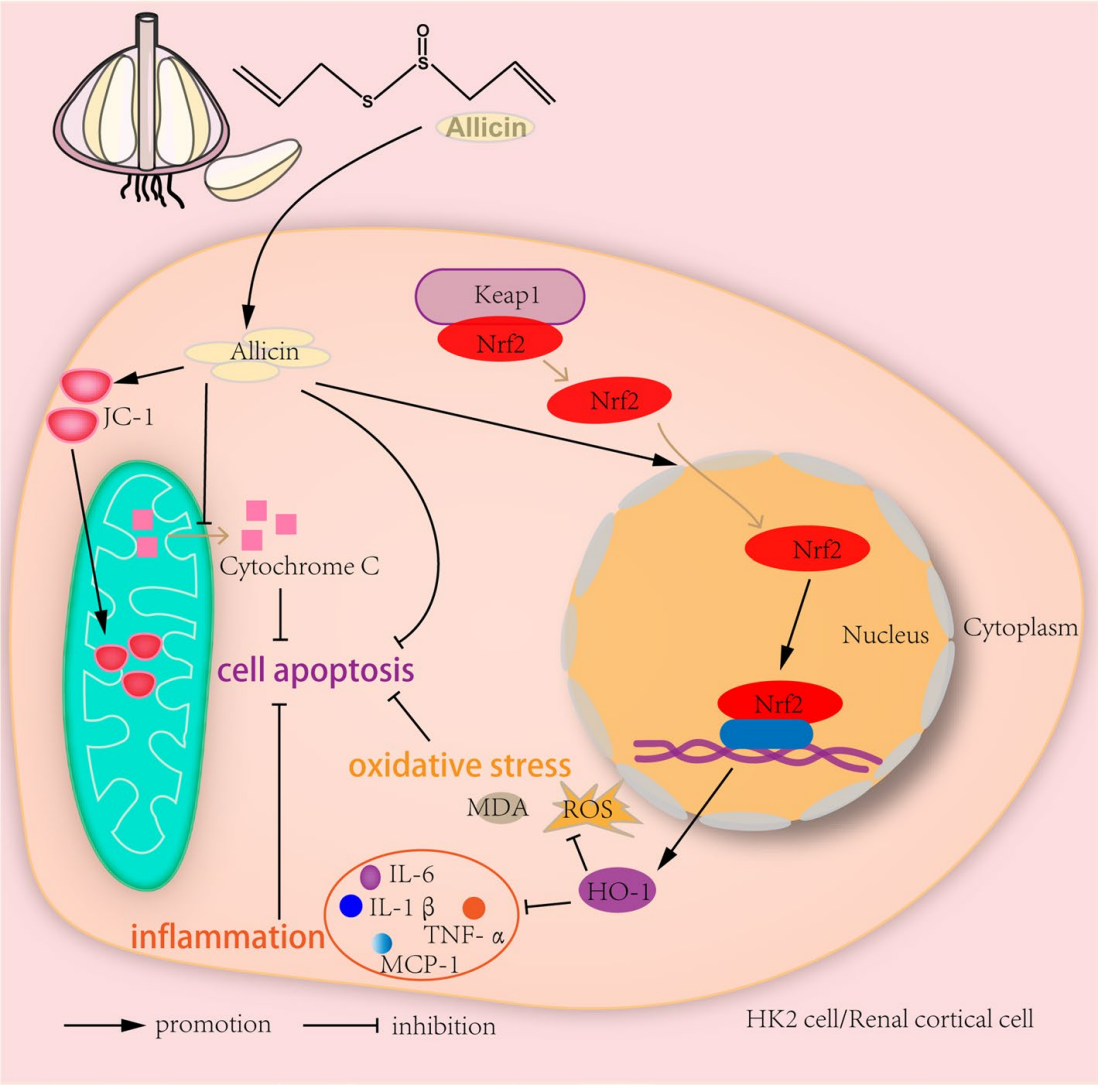
Mechanism diagram of allicin inhibiting oxidative stress, inflammation and apoptosis. Allicin promoted nuclear translocation of Nrf2 and the expression of HO-1, followed by reducing the levels of IL-1β, IL-6, TNF-α, MCP-1, ROS and MDA. Allicin inhibited the release of cytochrome C from mitochondria to cytoplasm and restored mitochondrial dysfunction. Allicin also inhibited cell apoptosis. In conclusion, allicin inhibits inflammation, oxidative stress and cell apoptosis through Nrf2/HO-1 signaling pathway in sepsis-induced AKI

The alleviating effects of allicin on S-AKI were revealed for the first time in this study. We demonstrated the antioxidant and anti-inflammatory activity of allicin, as well as its ability to restore mitochondrial dysfunction and inhibit apoptosis both in vivo and in vitro. This study verified that allicin promoted nuclear translocation of Nrf2 and found that the inhibitory effect of allicin on inflammation, oxidative stress and cell apoptosis was reversed by ML385. These results showed that allicin inhibited S-AKI through Nrf2/HO-1 pathway (Fig. 8). The above findings provide a new reference drug for the treatment of S-AKI, and lay more experimental foundations for the pharmacological activity of allicin.

### Supplementary Information

Below is the link to the electronic supplementary material.

Supplementary file 1 Supplementary figure a. Levels of TNF-α, IL-1β and ROS as well as the activity of GSH-Px in HK2 cells with allicin and CDDO-Me treatment. b. Analysis of JC-1-labeled mitochondrial membrane potential by flow cytometry in LPS and allicin and CDDO-Me treated HK2 cells. c. Analysis of cell apoptosis by flow cytometry in HK2 cells with LPS and allicin and CDDO-Me treatments. All data were expressed as mean ± SD. * p< 0.05 and ** p<0.01, it was compared with LPS +allicin-20 group, n=3 in each group, and the number of technical replicates is 3.

## References

[1] Singer M, Deutschman CS, Seymour CW, Shankar-Hari M, Annane D, Bauer M (2016). The third international consensus definitions for sepsis and septic shock (Sepsis-3). JAMA.

[2] Angus DC, van der Poll T (2013). Severe sepsis and septic shock. N Engl J Med.

[3] Cecconi M, Evans L, Levy M, Rhodes A (2018). Sepsis and septic shock. Lancet.

[4] Poston JT, Koyner JL (2019). Sepsis associated acute kidney injury. BMJ.

[5] Russell JA, Singer J, Bernard GR, Wheeler A, Fulkerson W, Hudson L (2000). Changing pattern of organ dysfunction in early human sepsis is related to mortality. Crit Care Med.

[6] Koeze J, Keus F, Dieperink W, van der Horst IC, Zijlstra JG, van Meurs M (2017). Incidence, timing and outcome of AKI in critically ill patients varies with the definition used and the addition of urine output criteria. BMC Nephrol.

[7] Peerapornratana S, Manrique-Caballero CL, Gomez H, Kellum JA (2019). Acute kidney injury from sepsis: current concepts, epidemiology, pathophysiology, prevention and treatment. Kidney Int.

[8] Gotts JE, Matthay MA (2016). Sepsis: pathophysiology and clinical management. BMJ.

[9] El-Saber Batiha G, Magdy Beshbishy A, L GW, Elewa YHA, A AA-S, Abd El-Hack ME, et al. (2020) Chemical Constituents and Pharmacological Activities of Garlic (Allium sativum L.): A Review. Nutrients. 10.3390/nu12030872 10.3390/nu12030872 PMC7146530 32213941

[10] Li B, Zheng F, Chauvin JR, Pratt DA (2015). The medicinal thiosulfinates from garlic and Petiveria are not radical-trapping antioxidants in liposomes and cells, but lipophilic analogs are. Chem Sci.

[11] Borlinghaus J, Albrecht F, Gruhlke MC, Nwachukwu ID, Slusarenko AJ (2014). Allicin: chemistry and biological properties. Molecules.

[12] Zainal M, Mohamad Zain N, Mohd Amin I, Ahmad VN (2021). The antimicrobial and antibiofilm properties of allicin against Candida albicans and *Staphylococcus aureus*—a therapeutic potential for denture stomatitis. Saudi Dent J.

[13] Zhou Y, Li X, Luo W, Zhu J, Zhao J, Wang M (2022). Allicin in digestive system cancer: from biological effects to clinical treatment. Front Pharmacol.

[14] Gu X, Wu H, Fu P (2013). Allicin attenuates inflammation and suppresses HLA-B27 protein expression in ankylosing spondylitis mice. Biomed Res Int.

[15] Qian YQ, Feng ZH, Li XB, Hu ZC, Xuan JW, Wang XY (2018). Downregulating PI3K/Akt/NF-kappaB signaling with allicin for ameliorating the progression of osteoarthritis: in vitro and vivo studies. Food Funct.

[16] Pandurangan AK, Ismail S, Saadatdoust Z, Esa NM (2015). Allicin alleviates dextran sodium sulfate- (DSS-) induced ulcerative colitis in BALB/c mice. Oxid Med Cell Longev.

[17] Shen N, Cheng A, Qiu M, Zang G (2019). Allicin improves lung injury induced by sepsis via regulation of the toll-like receptor 4 (TLR4)/myeloid differentiation primary response 88 (MYD88)/nuclear factor kappa B (NF-kappaB) pathway. Med Sci Monit.

[18] Wang X, Zhang C, Chen C, Guo Y, Meng X, Kan C (2018). Allicin attenuates lipopolysaccharide-induced acute lung injury in neonatal rats via the PI3K/Akt pathway. Mol Med Rep.

[19] Vimal V, Devaki T (2004). Hepatoprotective effect of allicin on tissue defense system in galactosamine/endotoxin challenged rats. J Ethnopharmacol.

[20] Arellano-Buendia AS, Castaneda-Lara LG, Loredo-Mendoza ML, Garcia-Arroyo FE, Rojas-Morales P, Arguello-Garcia R (2020). Effects of allicin on pathophysiological mechanisms during the progression of nephropathy associated to diabetes. Antioxidants.

[21] Arellano Buendia AS, Tostado Gonzalez M, Sanchez Reyes O, Garcia Arroyo FE, Arguello Garcia R, Tapia E (2018). Immunomodulatory effects of the nutraceutical garlic derivative allicin in the progression of diabetic nephropathy. Int J Mol Sci.

[22] Huang H, Jiang Y, Mao G, Yuan F, Zheng H, Ruan Y (2017). Protective effects of allicin on streptozotocin-induced diabetic nephropathy in rats. J Sci Food Agric.

[23] Zhang Y, Yao HP, Huang FF, Wu W, Gao Y, Chen ZB (2008). Allicin, a major component of garlic, inhibits apoptosis in vital organs in rats with trauma/hemorrhagic shock. Crit Care Med.

[24] Nath M, Agarwal A (2020). New insights into the role of heme oxygenase-1 in acute kidney injury. Kidney Res Clin Pract.

[25] Mohamed AF, Safar MM, Zaki HF, Sayed HM (2017). Telluric acid ameliorates endotoxemic kidney injury in mice: involvement of TLR4, Nrf2, and PI3K/Akt signaling pathways. Inflammation.

[26] Schabbauer G, Tencati M, Pedersen B, Pawlinski R, Mackman N (2004). PI3K-Akt pathway suppresses coagulation and inflammation in endotoxemic mice. Arterioscler Thromb Vasc Biol.

[27] Li D, Liang H, Li Y, Zhang J, Qiao L, Luo H (2021). Allicin alleviates lead-induced bone loss by preventing oxidative stress and osteoclastogenesis via SIRT1/FOXO1 pathway in mice. Biol Trace Elem Res.

[28] Yao W, Guo A, Han X, Wu S, Chen C, Luo C (2019). Aerosol inhalation of a hydrogen-rich solution restored septic renal function. Aging.

[29] Guerrero-Hue M, Rayego-Mateos S, Vazquez-Carballo C, Palomino-Antolin A, Garcia-Caballero C, Opazo-Rios L (2020). Protective role of Nrf2 in renal disease. Antioxidants.

[30] Legrand M, Bell S, Forni L, Joannidis M, Koyner JL, Liu K (2021). Pathophysiology of COVID-19-associated acute kidney injury. Nat Rev Nephrol.

[31] Wang Q, Yao YM, Wang WJ, Xian LM, Dong N, Xu S (2007). Effect of Xuebijing injection on renal high mobility group box-1 protein expression and acute kidney injury in rats after scald injury. Zhongguo Yi Xue Ke Xue Yuan Xue Bao.

[32] Zhang J, Yang S, Chen F, Li H, Chen B (2017). Ginkgetin aglycone ameliorates LPS-induced acute kidney injury by activating SIRT1 via inhibiting the NF-kappaB signaling pathway. Cell Biosci.

[33] Li H, Chen W, Chen Y, Zhou Q, Xiao P, Tang R (2019). Neferine attenuates acute kidney injury by inhibiting NF-kappaB signaling and upregulating klotho expression. Front Pharmacol.

[34] Tang JL, Xin M, Zhang LC (2022). Protective effect of astragalus membranaceus and Astragaloside IV in sepsis-induced acute kidney injury. Aging.

[35] Liu J, Zhao N, Shi G, Wang H (2020). Geniposide ameliorated sepsis-induced acute kidney injury by activating PPARgamma. Aging.

[36] Ma Q (2013). Role of nrf2 in oxidative stress and toxicity. Annu Rev Pharmacol Toxicol.

[37] Ahmed SM, Luo L, Namani A, Wang XJ (1863). Tang X (2017) Nrf2 signaling pathway: pivotal roles in inflammation. Biochim Biophys Acta Mol Basis Dis.

[38] Jiang T, Harder B, Rojo de la Vega M, Wong PK, Chapman E, Zhang DD (2015). p62 links autophagy and Nrf2 signaling. Free Radic Biol Med.

[39] Zhang X, Zhu Y, Zhou Y, Fei B (2020). Activation of Nrf2 signaling by apelin attenuates renal ischemia reperfusion injury in diabetic rats. Diabetes Metab Syndr Obes.

[40] Ryter SW, Choi AM (2016). Targeting heme oxygenase-1 and carbon monoxide for therapeutic modulation of inflammation. Transl Res.

[41] Gozzelino R, Jeney V, Soares MP (2010). Mechanisms of cell protection by heme oxygenase-1. Annu Rev Pharmacol Toxicol.

[42] Bolisetty S, Zarjou A, Agarwal A (2017). Heme oxygenase 1 as a therapeutic target in acute kidney injury. Am J Kidney Dis.

[43] Lee S, Huen S, Nishio H, Nishio S, Lee HK, Choi BS (2011). Distinct macrophage phenotypes contribute to kidney injury and repair. J Am Soc Nephrol.

[44] Huen SC, Cantley LG (2017). Macrophages in renal injury and repair. Annu Rev Physiol.

[45] An C, Jiao B, Du H, Tran M, Zhou D, Wang Y (2022). Myeloid PTEN deficiency aggravates renal inflammation and fibrosis in angiotensin II-induced hypertension. J Cell Physiol.

[46] Jiao B, An C, Tran M, Du H, Wang P, Zhou D (2021). Pharmacological inhibition of STAT6 ameliorates myeloid fibroblast activation and alternative macrophage polarization in renal fibrosis. Front Immunol.

[47] Jiao B, An C, Du H, Tran M, Wang P, Zhou D (2021). STAT6 deficiency attenuates myeloid fibroblast activation and macrophage polarization in experimental folic acid nephropathy. Cells.

[48] An C, Jiao B, Du H, Tran M, Song B, Wang P (2023). Jumonji domain-containing protein-3 (JMJD3) promotes myeloid fibroblast activation and macrophage polarization in kidney fibrosis. Br J Pharmacol.

[49] Hastings JE (1986). Primary health care in Canada. Salud Publica Mex.

[50] Bat-Chen W, Golan T, Peri I, Ludmer Z, Schwartz B (2010). Allicin purified from fresh garlic cloves induces apoptosis in colon cancer cells via Nrf2. Nutr Cancer.

[51] Mocayar Maron FJ, Camargo AB, Manucha W (2020). Allicin pharmacology: common molecular mechanisms against neuroinflammation and cardiovascular diseases. Life Sci.

[52] Gruhlke MC, Slusarenko AJ (2012). The biology of reactive sulfur species (RSS). Plant Physiol Biochem.

[53] Nadeem MS, Kazmi I, Ullah I, Muhammad K, Anwar F (2021). Allicin, an antioxidant and neuroprotective agent, ameliorates cognitive impairment. Antioxidants.

[54] Schafer FQ, Buettner GR (2001). Redox environment of the cell as viewed through the redox state of the glutathione disulfide/glutathione couple. Free Radic Biol Med.

[55] Kensler TW, Wakabayashi N, Biswal S (2007). Cell survival responses to environmental stresses via the Keap1-Nrf2-ARE pathway. Annu Rev Pharmacol Toxicol.

[56] Kansanen E, Kuosmanen SM, Leinonen H, Levonen AL (2013). The Keap1-Nrf2 pathway: Mechanisms of activation and dysregulation in cancer. Redox Biol.

