# Supplementary figures and images for: Allicin ameliorates sepsis-induced acute kidney injury through Nrf2/HO-1 signaling pathway
Source: J Nat Med. 2023 Sep 5;78(1):53–67. doi: 10.1007/s11418-023-01745-3 (PMC10764392; doi:10.1007/s11418-023-01745-3)

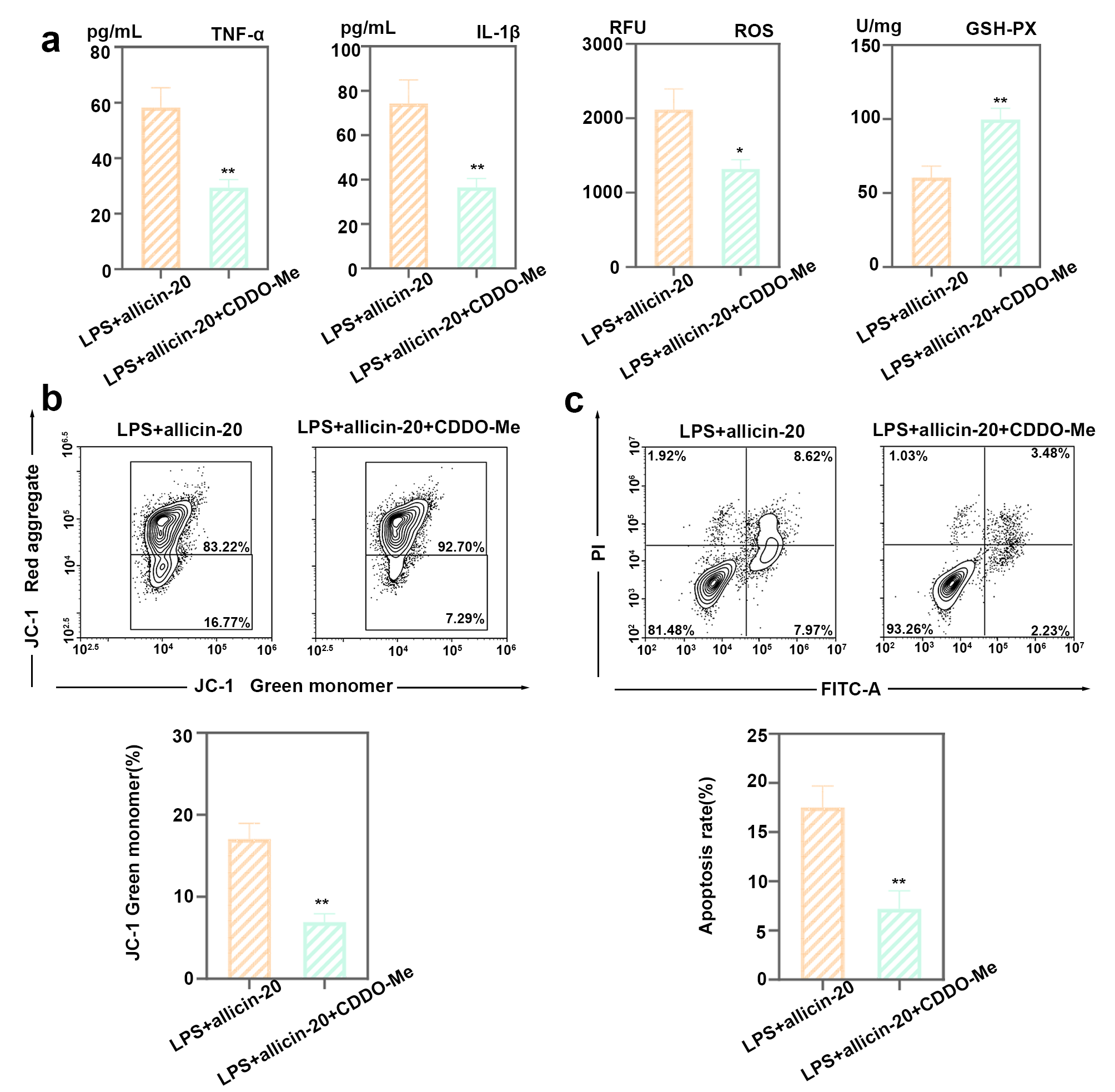

Supplement: Supplementary file 1 — Supplementary figure a. Levels of TNF-α, IL-1β and ROS as well as the activity of GSH-Px in HK2 cells with allicin and CDDO-Me treatment. b. Analysis of JC-1-labeled mitochondrial membrane potential by flow cytometry in LPS and allicin and CDDO-Me treated HK2 cells. c. Analysis of cell apoptosis by flow cytometry in HK2 cells with LPS and allicin and CDDO-Me treatments. All data were expressed as mean ± SD. * p< 0.05 and ** p<0.01, it was compared with LPS +allicin-20 group, n=3 in each group, and the number of technical replicates is 3. [file 11418_2023_1745_MOESM1_ESM.tif]
